# Time-Resolved Tomographic Quantification of the Microstructural Evolution of Ice Cream

**DOI:** 10.3390/ma11102031

**Published:** 2018-10-19

**Authors:** Jingyi Mo, Enyu Guo, D. Graham McCartney, David S. Eastwood, Julian Bent, Gerard Van Dalen, Peter Schuetz, Peter Rockett, Peter D. Lee

**Affiliations:** 1 Department of Mechanical Engineering, University College London, London WC1E 7JE, UK; graham.mccartney@nottingham.ac.uk (D.G.M.); peterrockett33@gmail.com (P.R.); 2 Research Complex at Harwell, RAL, Didcot OX11 0FA, UK; david.eastwood@manchester.ac.uk; 3 School of Materials Science and Engineering, Dalian University of Technology, Dalian 116024, China; eyguo@dlut.edu.cn; 4 The Manchester X-ray Imaging Facility, School of Materials, The University of Manchester, Manchester M13 9PL, UK; 5 Unilever R&D, Colworth MK44 1LQ, UK; Julian.Bent@unilever.com (J.B.); Gerard-van.Dalen@unilever.com (G.V.D.); Peter.Schuetz@unilever.com (P.S.)

**Keywords:** ice cream, microstructure, tomography, ice crystals, coarsening, soft solids

## Abstract

Ice cream is a complex multi-phase colloidal soft-solid and its three-dimensional microstructure plays a critical role in determining the oral sensory experience or mouthfeel. Using in-line phase contrast synchrotron X-ray tomography, we capture the rapid evolution of the ice cream microstructure during heat shock conditions in situ and *operando*, on a time scale of minutes. The further evolution of the ice cream microstructure during storage and abuse was captured using ex situ tomography on a time scale of days. The morphology of the ice crystals and unfrozen matrix during these thermal cycles was quantified as an indicator for the texture and oral sensory perception. Our results reveal that the coarsening is due to both Ostwald ripening and physical agglomeration, enhancing our understanding of the microstructural evolution of ice cream during both manufacturing and storage. The microstructural evolution of this complex material was quantified, providing new insights into the behavior of soft-solids and semi-solids, including many foodstuffs, and invaluable data to both inform and validate models of their processing.

## 1. Introduction

**Figure 1:**
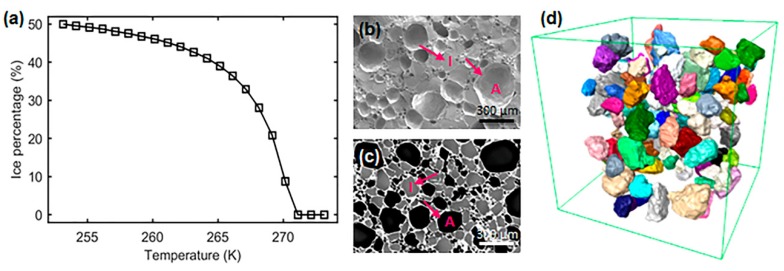
The characteristics of ice crystals in ice cream samples: (**a**) ice crystal volume fraction as a function of temperature; (**b**) a typical cryo-SEM image, showing air cells, A, and ice crystals, I; (**c**) 2D X-ray tomographic image of an ice cream sample. Note, the contrast was enhanced via edge-constrained diffusion filtering (300 iterations). Dark features are air cells, grey features are ice crystals and white regions are the unfrozen matrix; (**d**) 3D renderings of the ice crystals from X-ray tomographic images, providing a 3D view of the features. Here, the ice crystals are individually color rendered (after Reference [2]).

Ice cream is a widely consumed dairy product whose complex microstructure determines its texture and oral sensory perception. The main constituents of ice cream are air cells, ice crystals, finely dispersed (emulsified) fat globules, and a continuous unfrozen aqueous solution phase (containing sugars and other additives) and, as shown in Figure 1a, the ice crystal fraction varies markedly with temperature. Ice cream is known to have a complex microstructure which is a function of its formulation but also its thermal history during manufacture, storage, and handling. An important aim of any manufacturer is to ensure their process produces ice cream with a smooth texture which does not degrade to a grainy and coarse texture before the product is consumed and enjoyed. The size of the ice crystals is widely recognized to be a key factor in the perception of texture by consumers; larger crystals being, in general, undesirable [1].

It is thus crucial to be able to observe how the complex three-dimensional structure of ice cream evolves over time with changes in temperature. The traditional method of examination using cryo-SEM of extracted samples (Figure 1b) is capable of providing excellent spatial resolution. However, the contrast between the different phases can be limited and only a two-dimension image is captured of the complex three dimensional (3D) interconnected network of air cells, ice crystals, and unfrozen matrix. During the past decade, there has been a significant increase in the use of synchrotron computed tomography (sCT) to examine the evolution of structures over time in three dimensions as the temperature is changed (termed 4D, 3D plus time, tomography) and so sCT is, in principle, well suited to the study of ice cream microstructures. Indeed, very recent studies have demonstrated the power of 3D sCT for studying the complex microstructure of ice cream; Figure 1c,d. sCT permits quantitative measurements of different phases and allows the visualization of 3D connectivity. 

In the present paper, we will describe the application of 4D sCT to reveal the microstructural evolution in ice cream over a range of different timescales, namely that which takes place during the manufacturing process (short timescales, minutes) and storage over long timescales (days/weeks). We apply in situ, time-resolved sCT to reveal the microstructure evolution mechanisms during manufacturing; whereas we use an ex situ sCT methodology to provide time-resolved data during long-term ice cream storage.

The manufacturing process for ice cream introduces around 50 vol% of air cells so the typical make up of frozen ice cream, by volume, is 50% air cells, 30% ice crystals, 10–15% sugar solution matrix and the balance dispersed fat droplets and non-fat solids [1,3]. Ice cream is commonly manufactured in a scraped-surface freezer (SSF) in either a batch or continuous mode as described, for example, in Cook and Hartel and others [4,5,6,7,8]. The initial processes occurring in the SSF are likely to be the most important in determining ice crystal size and size distribution. In a typical SSF, the mix is rapidly cooled at the wall of a highly chilled cylindrical barrel and ice crystals nucleate and grow at, or close to, the wall and potentially into an undercooled liquid [9]. Rotating scraper blades break up the initially formed crystals and sweep them into the bulk of the mix (where the mean temperature is closer to 269 K to 267 K) causing the fragmentation and partial melting of the initial ice crystals. This process has clear similarities to the metallurgical process known as rheocasting or thixocasting [10]. At the exit of the SSF, about 50% of the water is frozen and the mix is cooled to about 267 K. The semi-solid product is typically pumped into a container for supply to the consumer and hardened in an air blast freezer. It is cooled to around 255 K over a period of around 120 min (0.1 K/min) [9] and then stored at or below this temperature. Ideally, the ice crystals will be kept as small as possible at the exit from the SSF but as the temperature drops in the freezer the original ice crystals grow and their volume fraction increases (Figure 1a). 

The texture and sensorial quality of ice cream are related to the size and connectivity of the air cells and ice crystals [1,11,12]. It is reported that the microstructural features vary in size considerably with typical values being air cells 20–150 μm, ice crystals 10–75 μm, fat globules ~0.5 μm with the unfrozen matrix phase being a continuous matrix [9]. Once ice cream is distributed to consumers it can undergo significant temperature variations typically in the range 258 K to 268 K and these will affect the ice crystal size and hence perceived texture [7]. Empirically, it is found that long-term storage above 258 K and/or thermal cycling degrades the ice cream’s quality due to coarsening of ice crystals and air cells. Below about 243 K, the matrix phase is transformed to a glass which stabilizes the microstructure but above these temperature, ice crystals are thermodynamically unstable due to the curvature effects and the kinetics of ice crystal coarsening can be quite rapid. The well-known Ostwald ripening phenomenon is a major factor for ice crystal coarsening although other processes such as physical agglomeration also affect the size distribution. Measurements of crystal size distribution have shown that crystal coarsening is very significant in the temperature range of 258 K to 268 K and also that temperature oscillations of only around ±1 K can significantly enhance the coarsening rate [4].

Whilst significant progress has been made in elucidating the process-structure-quality relationships of ice cream, a major limitation is the complex procedures needed to perform microstructural observations by cryo-SEM or transmission electron microscopy (TEM). These are post-mortem techniques [13], and provide only two-dimensional (2D) information on the surface that sliced through the sample [14,15,16,17]. Consequently, interpretation of microstructure evolution is subject to assumptions and opens to ambiguity. Recently, X-ray microtomography techniques have been employed to study 3D structures in food products and frozen food, including ice cream, but with somewhat limited success [7,18,19]. Pinzer et al. used a laboratory X-ray microCT in a cold room to investigate the long-term microstructural evolution of ice cream and quantified changes in air cell and ice crystal size during thermal cycles between −5 to −16 °C over a period of 24 h [18]. However, the resolution of the structure was a limitation with this instrument.

Ice cream falls into the category of soft solids (of which there are a number found in nature, including food products) and has similar behavior and properties to many man-made and natural semi-solids. The microstructure of many of these materials evolves with time, changing material properties ranging from rheology to yield strength to plasticity, and, in the case of foodstuffs, mouthfeel. One way of quantifying this microstructural evolution is time-resolved radiography [20,21] or tomography [22,23]. Soft solids found in nature include soils [24], shales [25], and magma [26], and food products such as chocolate [27]. In recent years, there has been a growing interest in the use of 4D tomography, specifically time-resolved synchrotron computed tomography (sCT), to perform non-invasive 3D structure studies of soft solids with high resolution. For example, Kareh et al. used in situ experiments to follow the evolution of semi-solid aluminum alloys under heating and loading conditions [28] whilst Karagadde et al., used in situ indentation to uncover a new fracture mechanism, the transgranular liquation cracking of semi-solid Al-Cu alloys [29].

The use of high brilliance synchrotron X-ray computed tomography (sCT), coupled with a precise cold stage, to study ice cream microstructures has recently been reported by the current authors. In this prior study, we obtained the three-dimensional quantification of microstructural changes arising from the thermal cycling at low heating and cooling rates over periods of weeks. This was undertaken to simulate storage and transport effects on the ice cream product [2]. However, the details of ice crystal development during the manufacturing stages such as those involving processing in a scraped-surface freezer and subsequent slower cooling as a block in an air blast freezer are of great interest but have yet to be studied in detail. 

In the present study, we used a bespoke cold stage (capable of controlling the sample temperature precisely between 233 K and 293 K with 0.1 K accuracy) and employed high resolution (in terms of both spatial and contrast) sCT to conduct 4D (3D plus time) studies on the microstructural evolution of ice cream samples undergoing different types of thermal histories. In the first set of experiments, the ice cream samples underwent fast heating and cooling cycles to simulate aspects of manufacture and were continuously scanned, in situ and *operando*, during the process. The second set of experiments involved monitoring the microstructural evolution over long timescales and so ex situ experiments could be used to capture the time-dependent evolution. Novel image-based quantification techniques were developed to precisely and robustly evaluate the structural characteristics in the ice cream samples. Variations in the size distribution of ice crystals and unfrozen matrix were followed during the thermal-induced microstructural evolution as these are recognized as indicators for changes in texture and oral sensory perception. 

## 2. Materials and Methods

### 2.1. Preparation of Ice Cream Samples

500 mL blocks of fresh ice cream containing 5% fat and 40% ice were prepared (Unilever R&D, Colworth, UK). Prior to the in situ tomographic experiments, a block of fresh ice cream was left at room temperature to melt and partially de-aerate. Kapton tubes with 3 mm inner diameters and 67 μm wall thickness (American Durafilm Co. Inc, Holliston, MA, USA) were filled with this liquid dairy mixture using a 5 mL syringe, followed by mounting them onto a specially designed cold stage which will be described in the next section.

For the ex situ samples, Kapton tubes were inserted into the central region of individual ice cream blocks and the dairy mixture was first subjected to blast freezing at 238 K and then stored at 248 K. Immediately before observation by synchrotron imaging, the Kapton tubes filled with ice cream were cut from the bulk samples on a dry ice bed (maintained at around 193 K) and then inserted into the specially designed cold stage. 

### 2.2. Cold Stage Experimental Setup and Thermal Cycling for Ex Situ and In Situ sCT

**Figure 2:**
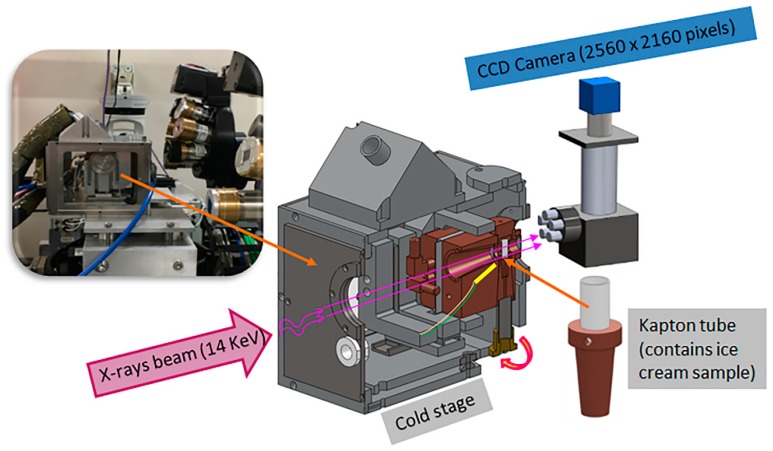
The experimental set-up in beamline I13, Diamond light source. The schematic shows the sectional view of the cold stage, the enlarged inset is the assembly of the ice cream sample, which consists of a 3 mm Kapton tube and the bottom copper mounts.

The cold stage assembly is shown schematically in Figure 2 and described in detail in our previous paper [2]. Both the ex situ and in situ sCT experiments were conducted on beamline I13-2 of the Diamond Light Source (DLS, Harwell, UK) using a pink beam. Details of the beamline set-up are described in the next section.

**Figure 3:**
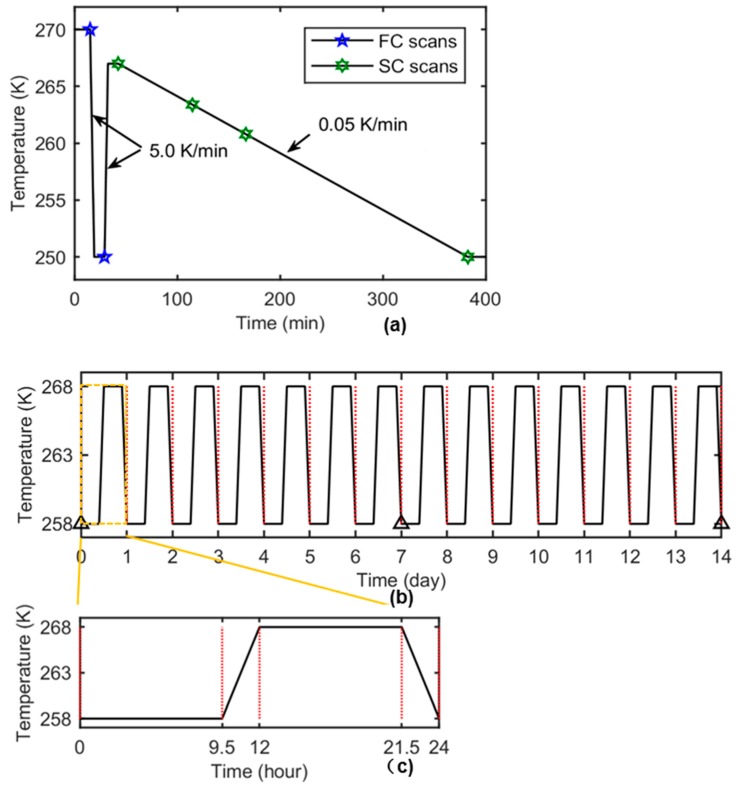
The thermal history of the samples during the in situ and ex situ experiments. (**a**) The overall thermal history of the in situ ice cream samples; the temperature points where the tomographic scans were reconstructed are indicated as blue pentagon and green hexagon markers. For FC, the images were reconstructed at 270 K and 250 K (blue pentagons). For SC, the reconstructed images were obtained at 266.8 K (SC0), 263.4 K (SC1), 260.8 K (SC2), and 250 K (SC3) (green hexagons). The cooling rates for FC and SC are 5.0 K/min and 0.05 K/min, respectively. (**b**) The overall thermal history of the ex situ ice cream samples, with triangular markers indicating the positions where the ice cream samples were extracted and scanned. (**c**) The zoom-in thermal profile showing a single ex situ thermal cycle.

In order to investigate processes relevant to the manufacture of ice cream, the in situ thermal cycling experiment was performed following the thermal cycles as described. A Kapton tube containing melted ice cream mixture was firstly mounted into the cold stage at the temperature of 270 K i.e., just below the melting temperature of the ice cream formulation. It was held at 270 K for 15 min and then its temperature was rapidly reduced to 250 K with a fast rate of cooling of 5 K/min (referred to as FC) and held at this temperature for 10 min. After that, the sample was heated to 267 K, at a heating rate of 5 K/min and held there for 10 min. Subsequently, it was cooled down from this temperature to 250 K at a slower rate of 0.05 K/min (referred to as SC). The overall thermal cycle for in-situ experiments is illustrated in the temperature versus time plot of Figure 3a. The fast cooling (FC) from 270 K allows one to study the initial ice crystal growth similar to that which might occur near the wall of an SSF. Fast reheating to 267 K approximates the behavior of ice crystals when swept into the bulk of the SSF. Finally, the SC regime from 267 to 250 K is regarded as representative of cooling of a block of ice cream mix in a blast freezer.

Blocks of ex situ ice cream samples were cycled between 258 K and 268 K multiple times, as shown in Figure 3b. In each daily routine (as shown in Figure 3c), the samples were held at 258 K for 9.5 h and then the temperature went up to 268 K in 2.5 h (with a rate of 0.067 K/min). After being held at 268 K for another 9.5 h, the sample temperature cooled back to 258 K with the same thermal rate (0.067 K/min) as before. This 24-h routine repeated for 7 times over one week (C7) and 14 times over two weeks (C14) before they were scanned using synchrotron X-ray tomography. The sample without any follow-on thermal treatment was referred to as C0.

### 2.3. Microstructural Characterization Using Synchrotron X-ray Computed Tomography (sCT)

Concurrently, with the thermal cycling of the sample, a series of X-ray tomographic images were acquired using sCT on I13-2 beamline at Diamond Light Source (Harwell, UK). This has a high flux undulator, producing a pink beam, with peak modes of narrow bandwidth (ca. 300 eV), with high and low bandwidth filters removing modes outside the energy ranging from 15 to 30 keV. This combined with the ca. 250 m beamline length provides excellent in-line phase contrast. A 2560 × 2160-pixel PCO Edge 5.5 CMOS camera optically coupled to a single crystal CdWO4 scintillator was used to record the projection images. The distance between the sample and the scintillator was optimized to be ~35 mm to achieve an optimum imaging quality. The final pixel size obtained was 0.81 µm for the scans.

During the in situ experiments, each tomographic run includes collecting 720 projections evenly spaced over a 180° rotation with the exposure time of 0.1 s. For FC, the scans were not recorded continuously due to the limitation of sampling rate relative to the fast cooling rate (5 K/min) and two images were acquired at the start and end points of the process, i.e., 270 K and 250 K, respectively, indicated as pentagons in Figure 3. For the same sampling limitation, there were no scans recorded during the fast heating (5 K/min). For the SC, the scans were recorded and pre-processed one by one and the 3D images were reconstructed at 266.8 K, 263.4 K, 260.8 K, and 250 K for analysis, as indicated as hexagons in Figure 3. These are referred to as SC0, SC1, SC2, and SC3 samples, respectively. For the ex situ experiments, each tomographic run includes 3601 projections over a 180° rotation with the same exposure time of 0.1 s for each projection.

### 2.4. Volume Data Reconstruction and Pre-Processing

The collected 2D radiographs/projections were stacked and converted into sinograms and in which any apparent continuous lines were removed by interpolation to reduce ring artifacts due to imperfections from the detector/camera. The sinograms were then used to reconstruct the volume slices using a filtered back projection (FBP) based algorithm. Because the ice cream samples were relatively low attenuating to the incident X-ray beam, the reconstructed volumes exhibited a relatively high level of noise. Therefore, the 3D volumes need to be filtered before any feature segmentation and quantification can be applied. Due to the microstructural differences in ex situ and in situ experiments, the data processing methods are slightly different.

For the ex situ experiments, a novel image processing strategy and reconstruction algorithm were used to achieve better quality images from the collected data; for details, see References [2,7]. A morphologically based method was used to quickly and robustly reduce the noise in the reconstructed volume of in situ data. First, 3D median filtering (3 × 3 × 3) was performed on all the reconstructed volumes. Then the volumes were cleared using a series of morphological operations. Both ex situ and in situ data were then binarized and labeled using global thresholding. All the volumes were subsequently checked visually, and any obvious segmentation imperfections were corrected manually using Avizo 9.4 (FEI Visualization Sciences Group, Mérignac, France).

### 2.5. 3D Based Quantification of Ice Crystal Dimensions

In the in situ study, an image analysis based quantification methodology of the ice crystal size in the ice cream samples was developed which is similar to the techniques for porous structure characterization for biomedical and geological samples [30]. Depending on the morphology of a porous structure, the pore size distribution can be obtained either by summarizing information of individually labeled components or estimated by measuring the variation of the interpenetrating volumes as a function of effective pore size. Here, as ice crystals appear as interconnected clusters, segmenting them into individual components is not appropriate. Therefore, we developed a method that provides an analysis similar to that of mercury intrusion porosimetry (MIP) to quantify the ice phase size distribution in the samples (see References [31,32]). In the MIP analysis, the pore size distribution within a porous sample can be determined from the cumulative volume of mercury liquid that has been forced into the pore space by externally applied high pressure. Here, we used a series of sampling spheres of varying diameter, and the size distribution in the ice crystal phase can be obtained by measuring the cumulative volume of ice crystal that can be reached by different sampling spheres.

The variation in accessible volume was used to estimate the ice crystal (or any segmented phase) size distribution, as follows:

1. A 3D distance map was first created by a Euclidean distance transform from the binarized image.
2. For the current samples, 13 sampling spheres with a diameter equally spaced between 5 µm and 40 µm were chosen to balance the quantification accuracy and computational expenses.
3. The radius of each sampling sphere (in voxels) was compared to the voxel intensities in the distance map, determining the centers of the spheres of a radius that can be completely placed within the ice phase.
4. A dilation algorithm was then applied to the voxels correlated to the sphere centers, using a spherical kernel of the same radius. The volume after dilation is the volume correlated to the sampling sphere.
5. Step 3–4 were repeated for all the sampling sphere dimensions chosen in step 2, providing a range of reachable volumes corresponding to increasing sphere diameters.
6. The volume fraction was calculated by dividing each volume by the total volume of the ice phase. Then an ice crystal size distribution can be calculated as the differential of the area under the cumulative volume percentage curve.

For the ex situ study, the reconstructed volumes from the data were cropped into a smaller volume, followed by the microstructural quantification in 3D. The three-dimensional rendering of the features, as well as the quantification of the ice crystals, was performed by manually using Avizo 9.4 (FEI Visualization Sciences Group, Mérignac, France). For more details, see References [2,7].

## 3. Results and Discussion

### 3.1. In Situ: Microstructural Evolution—2D Images

First, a tomographic scan was collected at 270 K prior to the onset of fast cooling. Except for air cells, it was not possible to resolve structural features in the material at this temperature. This indicates that it was above the freezing point of the ice cream composition under investigation and additionally significant supercooling is required before ice crystal nucleation occurs.

**Figure 4:**
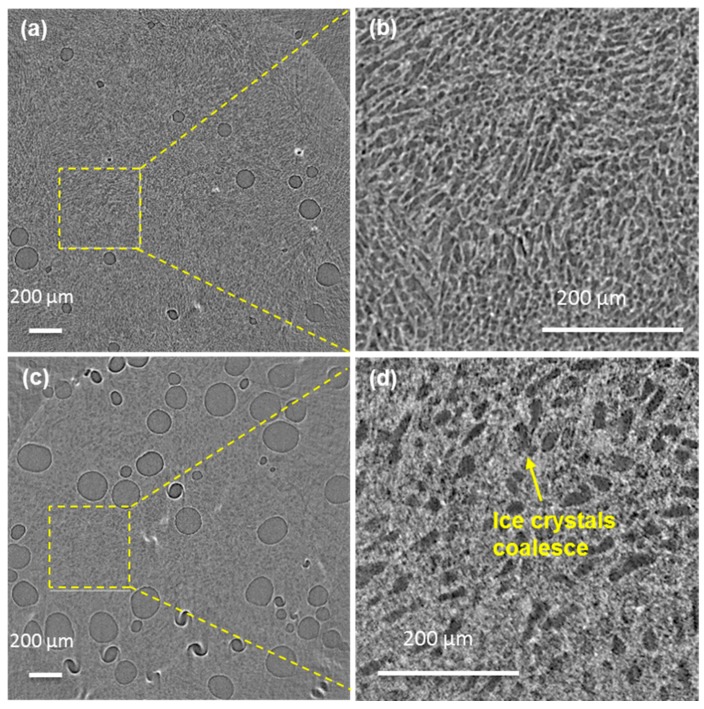
The reconstructed 2D tomographic slices. (**a**,**b**) Following fast cooling (FC) to 250 K. (**c**,**d**) Following heating to 266.8 K and holding for 10 min. In (a), the large circular dark areas with thin brighter boundaries are air cells and the matrix comprises of a dark contrast for the ice crystals and a brighter contrast for the unfrozen material. Zoom-ins of the areas highlighted by yellow squares in (**a**,**c**) are shown in (**b**,**d**) respectively.

Figure 4a,b show representative 2D tomographic image slices recorded after the fast cooling from 270 to 250 K and Figure 4c,d show representative image slices following the fast heating at 5 K/min from 250 to 267 K and holding at that temperature for 10 min. The main features evident at low magnification in Figure 4a,c are the light grey contrast circular features which are low X-ray attenuation air cells introduced by sample handling and imaging. These are embedded in a material with a fine scale microstructure showing grey/white contrast. At higher magnification (Figure 4b,d), the details of this matrix material’s microstructure are revealed. In Figure 4b, following fast cooling to 250 K, the microstructure comprises a high volume fraction of ice crystals (grey) with a lighter contrast continuous phase surrounding the crystals; the latter is the unfrozen matrix phase comprising a concentrated sucrose solution, fat globules, and solids. The ice crystals have a range of sizes and are apparently lozenge-shaped with dimensions of the order of 5 to 10 μm, with domains of aligned crystals. In Figure 4d, following fast heating from 250 K to 267 K, it is clear that the ice crystals occupy a significantly smaller volume fraction than at 250 K. Furthermore, the dimensions of the ice crystals have increased markedly following the fast heating and short, 10 min, holding time at 267 K. The coarsening could be due to factors such as Ostwald ripening and physical agglomeration during heating and holding. In Ostwald ripening, the driving force is the difference in solubility between the polydisperse particles of different curvatures. This solubility difference establishes a concentration gradient between the smaller and the larger particles, which leads to the growth of the larger particles at the expense of the smaller ones, the solute being transported through the unfrozen phase. It should also be noted that the volume fraction of air cells could potentially increase due to the photochemical cracking by the X-rays (Figure 4a,c) [33].

**Figure 5:**
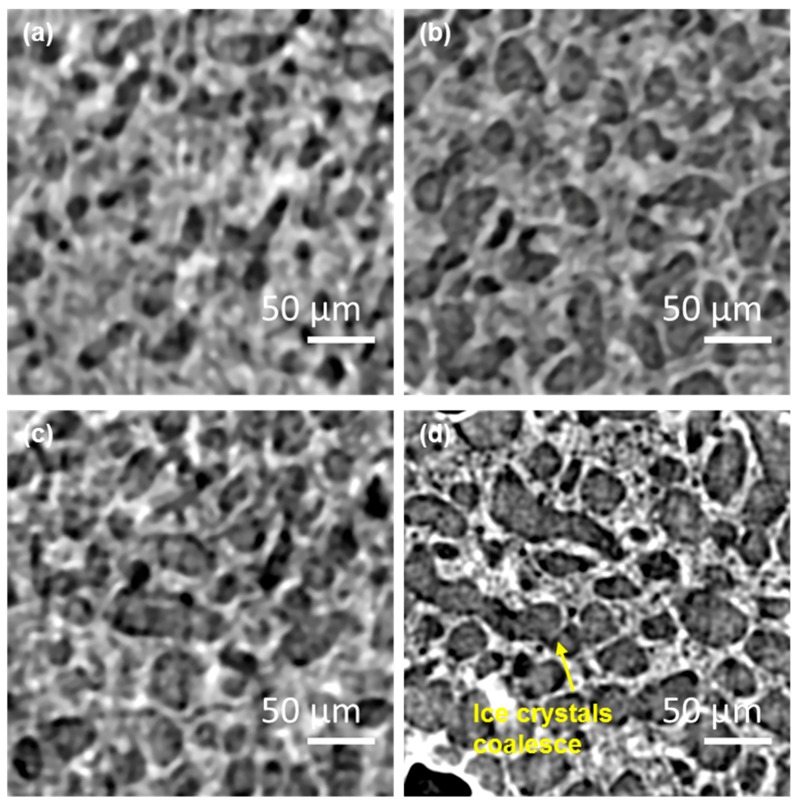
The 2D reconstructed tomographic slice showing the microstructural evolution of ice crystals during the slow cooling regime at the following temperatures: (**a**) 266.8 K, (**b**) 263.4 K, (**c**) 260.8 K and (**d**) 250 K. Note, for images taken at 260 K and above, coarsening occurs during image acquisition, blurring the image.

Figure 5a–d show the 2D tomographic slices recorded at 266.8 K, 263.4 K, 260.3 K, and 250 K respectively during the slow cooling (SC) regime (0.05 K/min) following a 10 min hold at 267 K. During this regime, it is obvious that ice crystals grow in size and the morphologies became more spherical than those at 267 K supporting the proposed mechanisms of Ostwald ripening and physical agglomeration. Moreover, the ice crystal volume fraction increases significantly and the specific interface area (S_s_) which is given by the interfacial area between ice (solid) and unfrozen matrix (liquid), denoted as A, divided by the total enclosed volume of ice (solid), denoted as V_s_, i.e., $S_{s}=\frac{A}{V_{s}}$, decreases during slow cooling and solidification (Table 1). There is also evidence, from the morphological features, that some of the ice crystals appear to have coalesced (see coalesced structure highlighted by an arrow in Figure 5d).

### 3.2. In situ: 3D Microstructural Evolution as a Function of Temperature and Time

**Figure 6:**
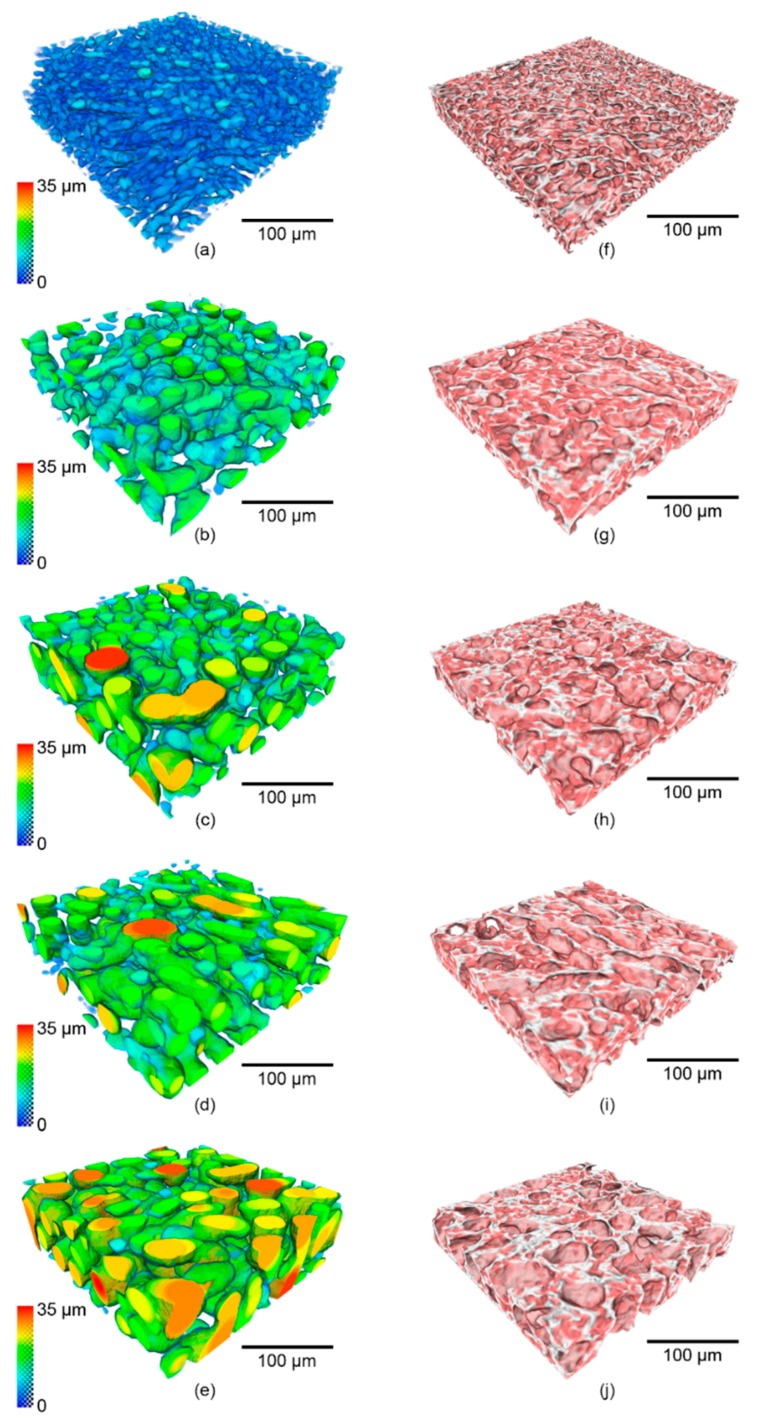
The 3D morphological evolution of representative volumes of (**a**–**e**) ice crystals and (**f**–**j**) the unfrozen matrix during the in situ experiment. (**a**,**f**) show the ice crystals and un-frozen matrix after fast cooling (FC) and holding at 250 K. During the slowing cooling (SC) period, the ice crystals, and un-frozen matrix are shown for the following temperatures: (**b**,**g**) 266.8 K, (**c**,**h**) 263.4 K, (**d**,**i**) 260.8 K, and (**e**,**j**) 250 K respectively. In (**a**–**e**), the color of the renderings correlates with the 3D local thickness of the ice crystals.

The 3D rendering of ice crystals and un-frozen matrix from representative regions of the same size are shown in Figure 6. In agreement with the 2D observations, the ice crystals are fine after the FC stage (Figure 6a) and after the fast heating and holding (Figure 6b), the small ice crystals have transformed into much larger ones. The ice crystal size continues to increase during the slow cooling period (Figure 6b–e). The color-coded local thickness of the ice crystals in Figure 6a–e allows us to better visualize the increase of the ice crystal thickness during the experiment (see quantification in the next section). The 3D interconnected volume of the unfrozen matrix is shown in Figure 6f–j. It appears that the wall thickness of the unfrozen matrix tended to increase upon heating (from 250 K to 267 K), whilst the thickness decreases as the temperature falls and the volume fraction of the ice increases in line with Figure 1a.

**Figure 7:**
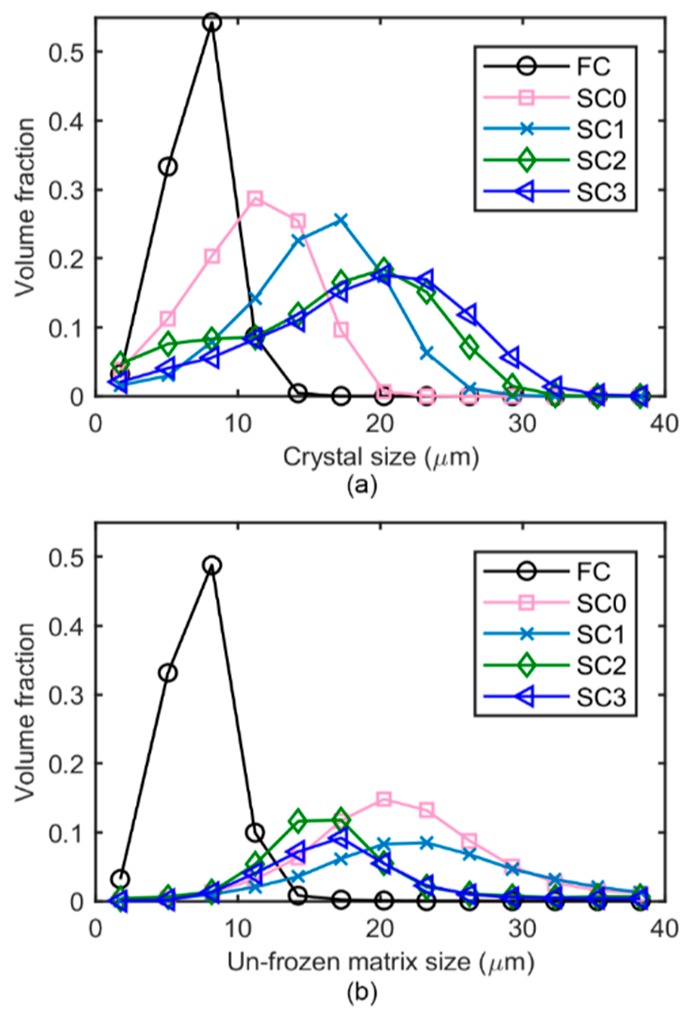
The size distributions of (**a**) the ice crystals and (**b**) the unfrozen matrix in the ice cream sample after fast cooling to 250 K (FC), and then after the fast reheating and holding process at 266.8 K (SC0), and during the slow cooling process at 263.4 K (SC1), 260.8 K (SC2), and 250 K (SC3), respectively.

The size distributions of ice crystals and the unfrozen matrix, obtained from the 3D accessible volume method, are shown in Figure 7. As expected from the 2D tomographic slices (Figure 4 and Figure 5) and the 3D reconstructed volume rendering (Figure 6), after the FC stage the ice crystals were very fine with a modal size of 8.2 µm and a range from around 1 to 15 μm. When the temperature was raised from 250 K to 267 K at 5 K/min and held for 10 min, the modal value of the size distribution increased to 11.2 µm. The distribution curve shifted to the right with a wider distribution ranging from 1–26 µm, which suggests the melting of smaller ice crystals and the growth of larger crystals at high temperature accompanying an overall decrease in ice volume with the increasing temperature. During slow cooling at a rate of 0.05 K/min, the modal size increased dramatically to 17.2 µm at 263.4 K (SC1) and to 20.2 µm at 260.8 K (SC2). Once the temperature had dropped below 263 K, the changes in size distribution at this cooling rate were not significant, which indicates that continued cooling below 263 K does not have a significant influence on the size of ice crystals over the timescale of the present experiments. It is presumably due to the slow molecular thermal diffusion at low temperatures, resulting in a reduced rate of coarsening.

In a complementary manner, the effects of the thermal history on the unfrozen matrix were also quantified. Figure 7b shows the size distribution of the unfrozen matrix at different temperatures, quantified using the same method as that employed for ice crystals (see Section 2.5). The unfrozen matrix is a complex 3D network that maintains the integrity of the ice cream structure. The 3D volume rendering in Figure 6f–j show that the unfrozen matrix appeared thicker between the ice crystals during the heating regime, whilst it became thinner as the sample cooled down from 267 K to 250 K, due to the melting of the ice crystals during the heating and recrystallization during cooling.

These qualitative observations are supported by the numerical data. The unfrozen matrix at 267 K (20.2 µm) after heating is much greater in thickness than that measured at 250 K (8.2 µm), which supports the idea that small crystals were melting into the unfrozen matrix as the temperature increased. During the SC regime, a trend of decreasing size of the unfrozen matrix is observed when the temperature of the sample was slowly cooled down to 250 K. This is presumably due to the growth increasing volume fraction of ice crystals, and is consistent with our quantification for the overall increased size of the ice crystal.

The morphological changes of ice crystals are accompanied by changes in the total ice fraction and specific interface area (S_s_) as revealed in mm^−1^ when cooled down to 250 K.

Table 1. Both the volume fraction and specific interface area (S_s_) of ice crystals decreased during heating and holding, which supports the observation of the partial melting and coarsening of ice crystals. During this period, the needle-shaped ice crystals at 250 K became more spherical after the heating regime (clearly to minimize the interfacial free energy). The fraction of ice decreased significantly from 38% at 250 K to 28% 267 K, which is consistent with the melting phenomenon while heating. In the SC regime, we noted that the increase of the ice volume fraction with freezing is not completely monotonic. One possible reason for this is the relative movement of the entire volume during cooling, which might change the features that are measured.

The S_s_ of ice crystals first decreased dramatically from 599 to 301 mm^−1^ during the fast heating and holding period. However, the morphology of the ice crystals showed only small changes during the SC regime, as indicated by the average S_s_ decreasing from 301 mm^−1^ at 267 K to 247 mm^−1^ at 263 K, and then continuously decreased to 193 mm^−1^ when cooled down to 250 K.

### 3.3. Ex Situ: Microstructural Evolution of Ice Crystals Following Long-Term Thermal Cycling

**Figure 8:**
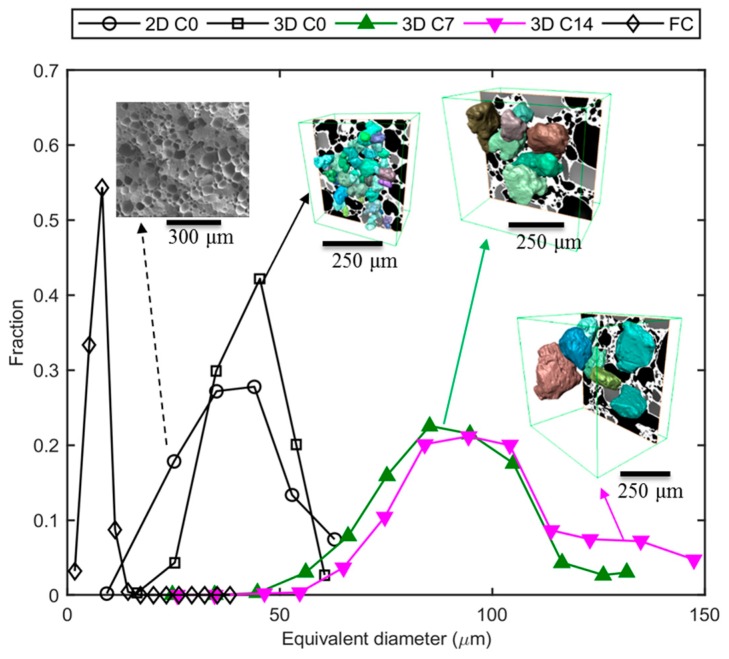
The size distributions of ice crystals in the ice cream samples following the FC stage from the in situ experiment, and C0, C7, C14 samples from the ex situ experiment. For the ex situ experiments, the morphology of the ice crystals is shown in the inserts as 3D color renderings of individual ice crystals. Additional data are taken from Reference [7].

Apart from the dynamic evolution mechanism of the individual features in ice cream which were elucidated in the in situ study, an examination of the microstructural evolution over long timescales (days/weeks) was also performed. The results are summarized in Figure 8 and reported in more detail in a previous paper [7]. After the thermal cycling from 258 to 268 K for 7 days, the size of ice crystals increased from a modal value of 45 µm in the C0 sample to a modal value of 85 µm in the C7 sample leading to coarsened ice structures and an ice cream that is likely to have a less desirable perception of texture for consumers. The size of the ice crystals was found to continuously grow even after 14-day long cycles. However, the rate of growth dramatically decreased after 7 cycles (7-day sample), with the size of ice crystals increasing only by ~10 µm from sample C7 to sample C14. It is possible that most of the surfaces of the ice crystals following 7 cycles are close to the size of the walls between the air cells. Therefore, air cells act as diffusion barriers, reducing Ostwald growth to being controlled by one-dimensional diffusion only in the plane of the wall. To demonstrate the morphological evolution in detail, a representative 3D rendering of ice crystals from the sCT data is superimposed in Figure 8.

Compared with the ex situ study, the size distribution obtained following the FC stage of the in situ work has a much smaller modal value of 8.2 µm. Such a fine scale microstructure is possible due to the fast cooling from the ice cream mix (liquid), which is different from the ex situ coarsened ice cream sample which was being “thermally abused” for a number of cycles between 258 and 268 K, i.e., the maximum temperature was well below the temperature of zero ice fraction (Figure 1a).

## 4. Conclusions

In the present study, we applied in situ and *operando* time-resolved synchrotron tomography in a bespoke cold stage to quantify the fast evolution of the different microstructural phases in ice cream during processing, including ice crystals and the unfrozen matrix. To capture the long-term microstructural evolution during storage, we used an ex situ sCT methodology.

The in situ experimental results in this study reveal that the coarsening of ice crystals was due to both Ostwald ripening and physical agglomeration during heating and cooling. This change in the ice crystals size and morphology strongly influences our sensory perception of ice cream’s taste. During the subsequent storage, we demonstrate that fluctuations in storage temperature can cause a partial-melting and recrystallization process, increasing the rate of coarsening. These processes were quantified, providing valuable data to both inform and validate models of the behavior of soft-solids.

## Figures and Tables

**Table 1:** The total volume fraction and specific interface area (S_s_) of ice crystals quantified by 3D tomography.

Cooling Rate	Temperature (K)	Ice Volume Fraction (%)	specific Interface Area (S_s_) (mm^−1^)
FC	250.0	38	599
SC0	266.8	28	301
SC1	263.4	40	247
SC2	260.3	36	233
SC3	250.0	46	193
